# The harmonium model and its unified system view of psychopathology: a validation study by means of a convolutional neural network

**DOI:** 10.1038/s41598-022-26054-9

**Published:** 2022-12-16

**Authors:** Linda A. Antonucci, Loredana Bellantuono, Johann Roland Kleinbub, Annalisa Lella, Arianna Palmieri, Sergio Salvatore

**Affiliations:** 1 Department of Translational Biomedicine and Neuroscience “DiBraiN”, University of Bari Aldo Moro, 70124 Bari, Italy; 2 Istituto Nazionale di Fisica Nucleare, Sezione di Bari, 70125 Bari, Italy; 3 Department of Philosophy, Sociology, Education and Applied Psychology, University of Padova, 35139 Padua, Italy; 4 Padova Neuroscience Center, University of Padova, 35129 Padua, Italy; 5 Department of Human and Social Science, University of Salento, 73100 Lecce, Italy

**Keywords:** Psychology, Computational science

## Abstract

The harmonium model (HM) is a recent conceptualization of the unifying view of psychopathology, namely the idea of a general mechanism underpinning all mental disorders (the p factor). According to HM, psychopathology consists of a low dimensional Phase Space of Meaning (PSM), where each dimension of meaning maps a component of the environmental variability. Accordingly, the lower thenumber of independent dimensions in the PSM, and hence its intrinsic complexity, the more limited the way of interpreting the environment. The current simulation study, based on a Convolutional Neural Network (CNN) framework, aims at validating the HM low-dimensionality hypothesis. CNN-based classifiers were employed to simulate normotypical and pathological cognitive processes. Results revealed that normotypical and pathological CNNs were different in terms of both classification performance and layer activation patterns. Using Principal Component Analysis to characterize the PSM associated with the two algorithms, we found that the performance of the normotypical CNN relies on a larger and more evenly distributed number of components, compared with the pathological one. This finding might be indicative of the fact that psychopathology can be modelled as a low-dimensional, poorly modulable PSM, which means the environment is detected through few components of meaning, preventing complex information patterns from being taken into account.

## Introduction

The taxonomic model of psychopathology relies on the assumption that there exist different forms of mental disease, and that each of them corresponds to a nosological category, like those reported in the Diagnostic and Statistical Manual of Mental Disorders, as well as in the International Classification of Disease^1,2^. The taxonomic model is helpful in specifying names, symptoms and diagnostic criteria for any given mental disease, and organizes all of them in multilevel groups, thus facilitating the communication among professionals involved in mental healthcare. Yet, studies have shown that the taxonomic model has limited clinical utility^3^. Moreover, the validity of such a paradigm (i.e., the extent to which a given diagnostic category could be considered a distinct and separate disease entity with clear boundaries like genetic markers, pathophysiology, neural substrates^4^) is poorly supported. On the one hand, symptoms belonging to the same diagnostic category occurring in different individuals may be explained by different causal mechanisms, thus suggesting high heterogeneity among individuals. On the other hand, many diagnostic categories overlap and may share biological features, indicating a lack of marked natural boundaries between them, and likely suggesting the existence of a continuum^5^. Thus, the empirical foundations underlying the assumption of the categorical structure of mental disorders, as well as the thresholds between diagnostic categories, must be considered weak.

Studies have highlighted the existence of substantial correlation among psychopathological characteristics, transversal to their taxonomy^6–9^. In recent years, this has led researchers and clinicians to hypothesize that psychopathology lies on a single *normal-abnormal* continuum, and that it would be worth focusing on the investigation of a potential underlying domain of human variation, of which the many mental disorders represent specific instances and manifestations^10^, rather than on the differences between categories. In other words, as for the g factors in cognitive sciences^11^, various authors have hypothesized the existence of a common factor of psychopathology (i.e., the p factor^12^), which would (i) explain covariance between different diagnostic categories, and (ii) account for individuals’ likelihood to develop any or all forms of psychopathological conditions^10^. Studies have so far shown that the p factor predicts mental disorders and behavioral problems^13–15^ also in longitudinal frameworks^16^, and preliminary evidence of its heritability has been recently reported^12,17^.

As an alternative to the taxonomic model of psychopathology^18,19^, the p factor framework has the potential of going beyond many of the previously described shortcomings of diagnostic categories. However, it should be noted that, so far, the evidence supporting the p factor hypothesis is robust, yet descriptive, and there is still no agreement on its clinical significance or on its underlying psychological mechanisms^20^.

The main explanations of the p factor have focused on three overarching dimensions: externalizing, internalizing and thought disorder^21^. The first and the second explanatory models identified the categories of externalization and internalization as dimensions at the basis of the p factor: the former includes a variety of externally-focused behavioral symptoms such as oppositionality, aggression, hyperactivity, attention and conduct problems, whereas the latter includes internally-focused symptoms including sadness/depression, anxiety, social withdrawal, and somatic complaints^21–27^. Despite their apparent phenomenological difference, externalizing and internalizing seem to be highly positively correlated with each another in children, adolescents, and adults (see for instance^28^). Specifically, according to some research work the p-factor is marked in internalizing facets^22,29^, whilst other studies suggested that it is mainly loaded by externalizing ones^25,30^.When the thought disorder dimension—consisting of psychotic characteristics ranging from unjustified irrational thoughts to hallucinations—has been added to the externalization and internalization, this third factor proved to provide the highest contribution to the explanation of the p factor^23,30–32^. The rationale of thought disorder as the underlying mechanism of the p factor is based on the evidence that these symptoms and their related psychological processes occur in many psychopathologies (e.g., in depression), rather than just in formal psychosis^33,34^. In addition to these three dimensions, a further hypothesis is that the functional mechanism of the p factor is based on poor constraint/control and impulsivity. This dimension proved to be strongly associated, on the one hand, with the externalizing one^19^, and, on the other hand, with the presence of deficits in cognitive functions (e.g. attention, concentration, mental control, visual-spatial processing speed and visual-motor coordination^30,35,36^).

Again, according to other authors, the p factor is due to the tendency to experience an unpleasant affective state^37^. In models of temperament and personality, such a dimension is labelled as "neuroticism" or "negative emotionality", and has been found to be associated with a wide range of mental and physical health problems^38^ and with both internalizing and externalizing dimensions of psychopathology in adults^8^. More generally, this viewpoint may be in line with those who have suggested relating the p factor to emotional intelligence, which in turn is seen as being associated with the ability to respond to social demands effectively^39^. Finally, a further interpretation of the p factor points out the rigidity of meaning making^40,41^.This interpretation is consistent with classical psychopathology views^42^ positing that mental disorders are the manifestations of intrinsically rigid, auto-protective and unable-to-change cognitive systems^41,43^.

Recently, our group has proposed a computational model of the mechanism underlying the p factor: the harmonium model. This paper reports a simulation study that provides further validation of the model. In what follows the harmonium model is briefly outlined; then, the design and findings of the study are described.

### The harmonium model and its computational implications

The harmonium model (HM)^40,41^ is consistent with the unifying view of psychopathology. The HM is based on the view of the environment as a dynamic field composed of an infinite set of co-occurring properties (e.g., colours, contours, temperature, pressure). Each of these properties has the potential to affect the state of the body and of the action. However, human cognitive systems are unable to map this potential infiniteness all together. Therefore, they have to extract meaningful and parsimonious sources of information that are able to map the core elements of the environment, though at the cost of a reduction in its dimensionality. It is only under this condition that the cognitive system can make inferences on the environmental field, and therefore regulate action.

This dimensionality reduction process resembles a Principal Component Analysis (PCA), in which human cognitive systems are called to cluster co-occurring states in synthetic dimensions of the whole environmental variability, each corresponding to a component of meaning.

At computational level, this process can be modelled in terms of the mathematical concept of phase space: the *Phase Space of Meaning (PSM)*^40^*.* According to this line of reasoning, each PSM dimension corresponds to a dimension of meaning that maps a component of the environmental variability. However, given the cognitive system’s constrained capacity of processing, not all PSM components can be taken into account at the same time: most of the very many potential components of the environmental variability are backgrounded, and only a few of them are simultaneously processed by the cognitive system. The modulation of the dimensionality of the PSM sets the number of components of the environment that the cognitive system will manage; this process is conceptualized through the representation of the mind as a harmonium which can extend and reduce the number of its active dimensions and thus its inherent complexity. In brief, a low-dimensional PSM corresponds to few components of the environment being taken into account, while a high dimensional PSM corresponds to high complexity of the foregrounded pattern of the environment.

A relevant characteristic of the way the PSM has been conceptualized^44^ is worth highlighting. Authors distinguish between *primary* and *secondary* dimensions of the PSM^10,41,45^. Primary dimensions consist of the basic embodied, generalized affect-laden meanings that shape the fundamental forms of interpretation of the experience (e.g., pleasant/unpleasant; active/passive). These fundamental forms of affective interpretation of the environment, as they have been modelled by several authors^46–48^, provide the global connotation of the overall environment that frames the rule-based, rational modes of processing its discrete characteristics, by means of differentiated, information-oriented meanings—e.g. ideas, concepts, scientific knowledge—that the culture system provides to meaning-makers. PSM secondary dimensions correspond to these differentiated meanings. Take a person encountering a stranger in an isolated place. First of all, she/he will activate a global basic affective connotation of the whole circumstance (e.g., friendly/unfriendly), that frames the subsequent processing of its specific characteristics. Thus, as it were, if the situation is felt to be unfriendly, the person foregrounds and therefore processes signals of potential threat coming from the other individual as well as the resources available to protect from it.

Primary dimensions are *basic* and *invariant*. Basic, in the sense that they comprise the core of any PSM—namely, they are the first to be activated in order to provide the global affective interpretation of the context of the experience. Invariant, in the sense that they are a few primitive (in the sense of foundational) classes of affect-laden meaning that work as a kind of universal embodied language at the boundary between biology and culture^44^. Thus, at the level of primary dimensions, PSMs are quite similar both within and between individuals. What makes PSMs vary are the secondary dimensions, which are potentially infinite. The more secondary dimensions are added to the primary dimensions, the higher the dimensionality of the PSM and the greater its specificity compared to other PSMs.

Based on these considerations, the HM holds that a low-dimensional PSM provides a computational model of the psychopathology. Indeed, a low-dimensional PSM describes a cognitive system whose way of processing the environment is heavily saturated by the core generalized affective meanings, with little room left to secondary dimensions, on which the detection of the more differentiated characteristics of the environment depends. To use an image, a low-dimensional PSM is like a person who experiences a wine just in terms of basic generalized characteristics (e.g., pleasant/unpleasant; cold/not cold), unable to recognize the nuances of its bouquet, for which the sommelier’s high-dimensional PSM is required. In the final analysis, according to the HM, psychopathology is a matter of a cognitive system constrained to process the experience only in terms of a few, basic dimensions of meaning, tending to be blind to the nuances and continuous environmental variability required for the more differentiated, secondary dimensions to be detected.

Incidentally, it is worth adding that the HM is consistent with the view of psychopathology as rigidity—that is, as the tendency to interpret and react to environmental stimuli in the same way even when such environmental stimuli are substantially different, and the response proves to be dysfunctional. Indeed, given that the primary dimensions are basic and invariant, a low-dimensional PSM, being saturated by them, corresponds to a way of making sense of experience that tends to reproduce itself invariantly, regardless of the characteristics of the environment. For the individual this results in a failure to grasp relevant elements of the environment she/he is relating to, and thus ultimately in potential maladjustment^45,49^.

The notion that psychopathology can be modelled at a computational level as a low-dimensional PSM is supported by a simulation study recently published by our group^10^. In the study, neural networks were trained on a visual classification task in order to show that the performance of deep learning routines is associated with the dimensionality of the phase space. The study hypothesis was that networks with high-dimensional internal dynamics would process novel information better than networks with low-dimensional internal dynamics. In the first part of the study, two identical networks (employing an unsupervised multi-layered deep belief algorithm) were trained on two different sets containing all 26 letters of the alphabet. The ‘high entropy’ set contained 27 font variations of each letter while the ‘low entropy’ one consisted of only 2 variations. Both networks were then tested using new graphical variations that were not present in the original sets as well as reduced image contrast, in order to mimic a difficult new environment. To ensure that results were not coincidental, in the second part of the study the same procedure was repeated 20 times, albeit, due to computational limitations, with only two letters (“A” and “M”). To evaluate the networks’ dimensional dynamics, a PCA was run on the windowed variance of the neurons’ activations, in order to represent phases of relatively homogeneous or turbulent activations in single neurons in time. A further PCA was then performed for each network’s neuron × time matrix to extract the dimensional structure underlying the neural network’s activity through time. Results showed that, as hypothesized, the networks trained with the high entropy set were more accurate than those trained with the low entropy set. Above all, the dimensionality of the networks was found to be associated with performance, with a negative effect concerning primary dimensions and a positive effect with secondary dimensions (primary and secondary dimensions were operationalized similarly to how it was done in the current study, see below). Overall, these results show that the limited variability of the low entropy learning environment resulted in overfitting the training data, preventing a generalisation of the learned patterns, and thus a more rigid behavior. On the contrary, better adaptivity was dependent on the network’s reliance on secondary dimensions.

However, it must be noted that these simulation findings have two major limitations. First, our previous study^10^ used a neural network architecture that did not allow the output of its inner layers to be analyzed. Accordingly, we focused on the internal dynamics of the neural network—i.e., the variation of any single neuron’s activation—rather than on the representation of the environment produced by such dynamics^10^. Therefore, the outcome of such research work provided only indirect support to the core tenet of the HM, which concerns the latter aspect, specifically—i.e., the low dimensionality of the PSM substantiating the psychopathological representation of the environment. Second, in the same study^10^ the two neural network’s modes of functioning—simulating the psychopathological and the normotypical cognitive processing respectively—were set up by means of a unidimensional manipulation of the training condition. Indeed, as already remarked, the low entropy and the high entropy conditions were obtained by modifying the amount of variability in the set of the training stimuli, using 2 vs 27 variations, respectively. The fact that the network trained in the low entropy condition performed worse than the one trained in the high entropy condition led us to conclude that the method adopted to simulate the cognitive processing underpinning psychopathology was effective. However, the unidimensional framework employed in Ref.^10^ is only partially consistent with the HM. This is so because the HM adopts a conception of the cognitive processes according to which psychopathology results from early exposure to low dimensional environments, namely environments that, regardless of their global magnitude of variability, are characterized by few components of variations^40^. In brief, the HM view of psychopathology as a matter of dimensionality implies that the onset of the psychopathology needs to be modelled in the same vein, namely as a matter of dimensionality of the developmental environment of the cognitive system. Therefore, one is led to conclude that the method to set the “psychopathological” neural network which is consistent with the HM framework is that which manipulates the dimensionality of the training environment, rather than the magnitude of its variability.

Thus, these previous findings^10^ highlight the urgency to obtain further empirical support via validation studies performed in more consistent conditions, for example by making the architecture and manipulation of the neural networks fit with the theoretical assumptions of the HM.

### Aims

This research proposes a further simulation study for testing the core hypothesis of the HM, namely the idea that the low-dimensional PSM is the computational equivalent of psychopathology. More particularly, the simulation carried out by the current study is aimed at providing a straightforward validation of the HM, overcoming the limitations of the previous simulation study^10^ highlighted above. The study pursues this purpose by: (i) employing a new deep learning architecture that enables the direct analysis of the representational output of the neural network, and (ii) adopting a dimensionality-based method to set the neural network simulating psychopathological cognitive processing, which is consistent with the HM framework.

As to the first aspect, we chose to use a Convolutional Neural Network (CNN)^50^, a state-of-the-art architecture that operationalizes computer vision tasks. This model is specifically appropriate to simulating cognitive processes, since CNN mimics the visual perception of a human being by identifying a spatial hierarchy among patterns: the first *convolutional layers* that characterize the CNN learn small patterns on a local scale, such as edges, while subsequent layers learn more extended patterns, made from the features provided by the previous layers’ output. In this way, increasingly complex and abstract visual concepts can be acquired by the model. Another distinctive feature of the CNN architecture, relevant for the current study, is its transparency, which makes it an ideal tool to validate the HM. While the majority of deep learning algorithms are commonly considered “black boxes” since they learn representations that are difficult to extract and present in a human-readable form, intermediate convolutional layers of a CNN provide, in their output, pixel activation patterns in different filters, that are simply representations of visual concepts, at different abstraction levels. CNNs therefore allow to visualize and analyze the response of these layers (intermediate activations) during the classification process of a new test instance, providing crucial indications on how the input is encoded and the information is elaborated. This characteristic of the CNN makes it highly appropriate to exploring how the HM works in different configurations.

As to the second aspect, we set the networks to simulate normotypical cognitive processing and pathological cognitive processing, respectively, by manipulating the dimensionality of the training task, rather than the quantity of variation in the set of stimuli. More particularly, stimuli were characterized by two dimensions (form and color)—in the low dimensional training condition (expected to set the “psychopathological” network), the two dimensions were fully associated with each other, while they were orthogonal in the high dimensional training condition (expected to set the “normotypical” network). It is worth highlighting the relevance of this new training setting. Indeed, it enables us to interpret the two kinds of networks compared by the study as simulating pathological and normotypical cognitive processing, respectively. In the previous study^10^, this interpretation emerged only a posteriori, based on the performance of the neural networks. Yet this criterion was descriptive and non-specific—a low performance neural network can be interpreted as the simulation of several types of critical cognitive processing (e.g. the biases characterizing the system information processing), not necessarily of the cognitive process underpinning the psychopathology. On the other hand, in the current study the interpretation of the low performance neural network as simulating psychopathology cognitive processing is not only based on the performance in the classification task; rather, it is grounded on a theoretical basis, namely on the fact that the “pathological” network was obtained by manipulating the training environment in a way that is fully consistent with how the HM framework models the onset of psychopathology.

### Hypothesis

With the previously described aims, we employed CNN to simulate a normotypical cognitive process and a pathological cognitive process, in order to model and compare their dimensionality. The CNN configurations simulating the normotypical and pathological cognitive processes respectively were built by varying the training stage to which the CNN was subjected. More specifically, to make the CNN simulate a normotypical cognitive process, it was subjected to a high-complexity training stage, while to obtain a pathological CNN it was subjected to a low-complexity training stage.

We assumed that the phase space required to describe the CNN’s functioning was the equivalent of the cognitive system’s PSM simulated by the CNN. Based on this assumption, we hypothesized that the functioning of the normotypical and the pathological CNNs show different phase spaces. More specifically, we hypothesized that the functioning of the normotypical CNN requires a higher dimensional phase space to be described than the pathological CNN. Moreover, we hypothesized that the normotypical CNN’s phase space shows a more even distribution between primary and secondary dimensions than the pathological CNN, the latter being characterized by a marked role played by primary dimensions with respect to secondary dimensions.

## Materials and methods

### Design of the study

**Figure 1:**
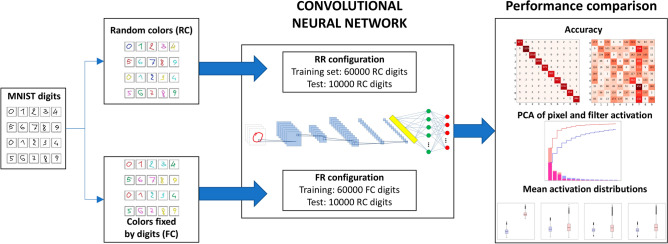
Workflow of the analysis. Colors are assigned to handwritten digits in the MNIST database^51^. A Convolutional Neural Network is trained with either randomly colored images, or with images whose colors are fixed by the digits therein. The test set is made in both cases by randomly colored images. We compare the two cases in terms of classification accuracy and confusion matrices, PCA of pixel activation and filter activation, and mean activation distributions.

The outline of the study is depicted in Fig. 1. We trained two CNN configurations to perform a well-established classification task, namely the recognition of handwritten digits^51^, which we modified by introducing a new feature, namely the color of the digits, occurring in six possibilities (red, cyan, green, magenta, yellow, blue). Color enters our model in two different training modes:

- in the *random color assignment* (RC) mode, color is fully independent from the geometrical patterns that characterize digits,
- in the *fixed color assignment* (FC) mode, well-determined colors are assigned to each digit in the training set (red for 0 and 1, cyan for 2 and 3, green for 4 and 5, magenta for 6 and 7, yellow for 8 and 9), thus inducing the algorithm to learn according to less flexible rules and oversimplified schemes.

We assumed that the different complexity of the training applied to the two CNN configurations—with the FC training being less complex than the RC—would lead to differentiate the capability of the CNNs to carry out the subsequent classification task. Indeed, one can expect that the less complex the training, the less the CNN would need to develop its classificatory capacity, and vice versa^10^.

The performances of the deep learning model, trained in both the aforementioned configurations, were then evaluated using an independent test set, in which the images of handwritten digits are colored following the RC mode. Specifically, the two case studies that will be analyzed and compared throughout the article, are defined as follows:

- *Random-color training set, random-color test set* (RR), in which colors in both the training and the test sets are assigned according to the RC mode;
- *Fixed-color training set, random-color test set* (FR), in which colors in the training set are assigned by the FC mode, and colors in the test set by the RC mode.

### Composition of training and test sets

To generate the images used to perform the CNN classification task, we started from the MNIST (Modified National Institute of Standards and Technology) database of handwritten digits, consisting of 70,000 greyscale images: 60,000 in the training set and 10,000 in the test set^51^. These images, size-normalized and centered in a $28\times 28$ pixel area, represent black digits (with different levels of grey) shown on a white background. In the present investigation, such images, initially encoded in one-channel tensors of $28\times 28\times 1$ dimensions, have been converted in the RGB format, characterized by *input depth* 3, thus becoming $28\times 28\times 3$ tensors. The conversion in the 3-channel RGB format is a necessary step to transform the images, which were initially in greyscale, by assigning them a color. In this framework, primary colors of the RGB model (red, green and blue), and their complementary counterparts (cyan, magenta and yellow), have been assigned to the digits in each image, keeping the original white background, according to one of the aforementioned criteria (RC mode or FC mode).

### Details of the convolutional neural network

A Convolutional Neural Network (CNN) is a Deep Learning algorithm that contains convolutional layers, which focus on local patterns by applying the same geometric transformation to different spatial locations in the image, as opposed to dense layers, in which only global patterns are learned. Due to the translation invariance of the representations learned by a convolutional layer and the possibility of a spatially hierarchical learning, CNN currently represents the state-of-the-art architecture for image recognition^50^.

A convolutional layer operates on a 3D input tensor, called *feature map*, whose first two dimensions indicate image height and width while the third one is the *input depth*. In the first CNN convolutional layer, the input depth coincides with the number of color channels of the image, equal, e.g., to 1 for greyscale and 3 for RGB. The convolution operation transforms the input feature map into an *output response map*, made of activation values, by applying local filters (in our case, covering $3\times 3$ pixel areas) with different patterns, whose number defines the *output depth*. The output response map yielded by a convolutional layer can then be used as an input feature map for a subsequent layer. Different convolutional layers in the CNN architecture are separated by max pooling layers, whose function is to downsample the input feature maps through $m\times m$ pixel blocks ($2\times 2$, in our case), and outputting the max value of each block.

**Figure 2:**
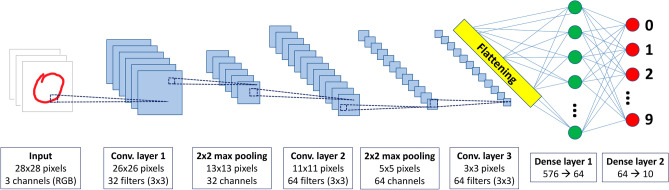
Scheme of the Convolutional Neural Network employed in our analysis.

The CNN employed in our analysis, displayed in Fig. 2, is made of the following layers:

- A first convolutional layer, that transforms a $28\times 28$ input image of depth 3 (number of channels in the RGB encoding) into a $26\times 26$ output response map of depth 32 (number of filters). This layer depends on 896 parameters.
- A first max pooling layer, that transforms each of the 32 output maps corresponding to a given filter from the first convolutional layer into a $13\times 13$ output image.
- A second convolutional layer, that transforms the $13\times 13$ input of depth 32 from the first max pooling unit into an $11\times 11$ output response map of depth 64. This layer depends on 18,496 parameters.
- A second max pooling layer, that transforms the $11\times 11$ output maps corresponding to the 64 filters from the previous convolutional layer into 5 × 5 output images.
- A third convolutional layer, that transforms the $5\times 5$ input of depth 64 from the second max pooling unit into a $3\times 3$ output response map of depth 64. This layer depends on 36,928 parameters.
- A first dense layer, that transforms a 576-dimensional input, obtained by flattening the output of the third convolutional layer, into a 64-dimensional output. This layer depends on 36,928 parameters.
- A second dense layer, that transforms the 64-dimensional output from the first dense layer into a 10-dimensional final output. This layer depends on 650 parameters.

### Training of convolutional neural networks

Once the CNN structure was defined, we set its working modes through the compilation step, consisting of specifying the following internal parameters:

- The *loss function*, used by the network as a feedback signal to monitor its performance on the training data, gaining information on the direction to optimize its weight values. In our study, we chose *categorical_crossentropy* as loss function, which measures the distance between two probability distributions, associated to the CNN predictions and the true labels, respectively. During the learning process on training data, *categorical_crossentropy* is minimized.
- The *optimizer*, representing the mechanism used by the network to adapt its configuration, based on the analyzed data and the loss function monitoring; we used the *rmsprop* optimizer.
- The *metric,* that quantifies CNN performances in the training and test phases. In our analysis, we chose accuracy as a metric, namely the fraction of correctly classified images.

The CNN was then trained on 60,000 instances, colored according to either the RC or the FC assignment rules. The classification accuracy on training images is 99.5% in the case of RC assignment and 99.9% in the case of FC assignment (the classification accuracy of test RC images will be discussed in the “Results” section).

### Comparison of CNN configurations simulating normotypical and pathological cognitive processes

To compare the performances and the computational features of the CNN classifiers in the RR (normotypical) and FR (pathological) cases, we followed the steps outlined below:

- *Classification accuracy comparison.* We analyzed the confusion matrices for the task of classifying the random-color MNIST database digits in the test set, using the CNNs trained in both the RR and FR modes, and evaluated the overall classification accuracy, representing the computational proxy of the effective fit between individual and environment in normotypical and pathological cognitive processes. This analysis served to check that the manipulation of the training phase was effective in making the CNN configurations simulate the normotypical and pathological cognitive systems, respectively
- *The phase space of the CNN dynamics*—*pixel level.* We modeled the phase space of the CNN dynamic of functioning by means of the Principal Component Analysis (PCA). The PCA enables to detect relevant directions in the space of convolutional layer features, i.e. patterns of covariation, each one mapped by a factorial component. Accordingly, any factorial space so obtained by PCA was interpreted as the phase space of the dynamic of functioning subjected to that PCA. Specifically, we started by applying PCA to the multivariate distributions of 10,000 points, representing each test sample, in the $N$-dimensional feature space of activation values corresponding to each pixel and each filter in a given layer. Thus, considering the size of output maps and the number of filters, we obtained $N=26\times 26\times 32= \mathrm{21,632}$ features for the first, $N=11\times 11\times 64=\mathrm{7,744}$ for the second, and $N=3\times 3\times 64=576$ for the third layer, respectively. We also constructed an overall distribution, in which point coordinates are the activation values for all ﻿29,952 pixels of all the layers together. The importance of each principal component is quantified by the ratio between the variance explained by that component and the total variance of the distribution. Following the previous simulation study carried out by our group^10^, we applied the following indices of dimensionality to the factorial spaces produced by the PCAs:
  - Weight of primary dimensions (WPD): the cumulative explained variance of the first two dimensions extracted by the PCA. The adoption of this criterion reflects the fact that many studies have found that there are two very basic dimensions of affective meanings (e.g. evaluation and dynamism^46^, pleasure/displeasure, and activated/deactivated^52^)
  - Weight of the secondary dimensions (WSD): cumulative explained variance of all factorial dimensions (other than the first two), having eigenvalue > 1 (the value of this threshold, was chosen following a Kaiser-like criterion^53^). The application of this criterion to the PCAs led to the selection of the following number of factorial dimensions:
    For the RR condition,15, 28, 19, 53 factorial dimensions of the PCAs applied to the first, second, third layer and overall distribution of pixel activations, respectively.
    For the FR condition,14, 30, 17, 52 factorial dimensions of the PCAs applied to the first, second, third layer and overall distribution of pixel activations, respectively.
  - Moreover, we added, as synthetic index of dimensionality, the number of factorial dimensions needed to explain 90% of the variance of the whole factorial space generated by the PCA (henceforth, D90). We adopted 90% as threshold in order to depict the dimensionality of the factorial space required to map a very large proportion of the whole explained variance, leaving apart the very marginal components (i.e. those associated with the last 10% of variance).
- *The phase space of the CNN dynamics*—*filter level.* We repeated the PCA analysis, changing the spatial scale from the single-pixel level to the filter level, by computing the mean activation value of each filter, defined as the mean of the activation values of its pixels. We evaluated the distributions of mean activation values on the filters of each convolutional layer, for the 10,000 test samples. In particular, each distribution includes the mean activation values of filters within a layer, produced by images in the test set; the overall distribution of mean pixel activations related to filters in the three convolutional layers together is also considered. In this framework, each of the 10,000 test instances is associated with the mean activation values of its image on the filters. This way, we obtained $N=32$ features for the first layer, $N=64$ for both the second and the third layer, and an overall distribution in which the 160 mean activation values for all layers together are associated to each test sample. The dimensionality of the factorial spaces obtained by the PCAs was measured by means of the same indexes described above, but WSD. Indeed, WSD was not appliable because, due to the low dimensionality of filter-level CNNs, no secondary dimension, or only 1, was identified.
- *Comparison of the mean activation value distributions (T-tests).* To further characterize and compare the behavior of pixel activation patterns throughout the normotypical and pathological CNN configurations, we investigated the distribution of mean activation values on the filters of each layer by means of two-sample T-tests, which allowed us to quantify the statistical difference between the RR and FR distribution pairs (alpha = 0.05, multiple comparisons corrected through False Discovery Rate—FDR^54^). We conducted four separate two-sample T-Tests having four different dependent variables: mean response values of the filters from the first, the second, and the third layer, as well as mean response values of filters from all the three layers together.

## Results

In this section we will outline results concerning the proxies which allow to characterize and compare the computational dynamics of the CNNs modeling the normotypical (RR) and pathological (FR) conditions: classification accuracy, principal components of the activation patterns of the CNN convolutional layers at both pixel and the filter levels, and comparison between the two CNN configurations’ distributions of mean activation values of test instances.

### Classification accuracy

**Figure 3:**
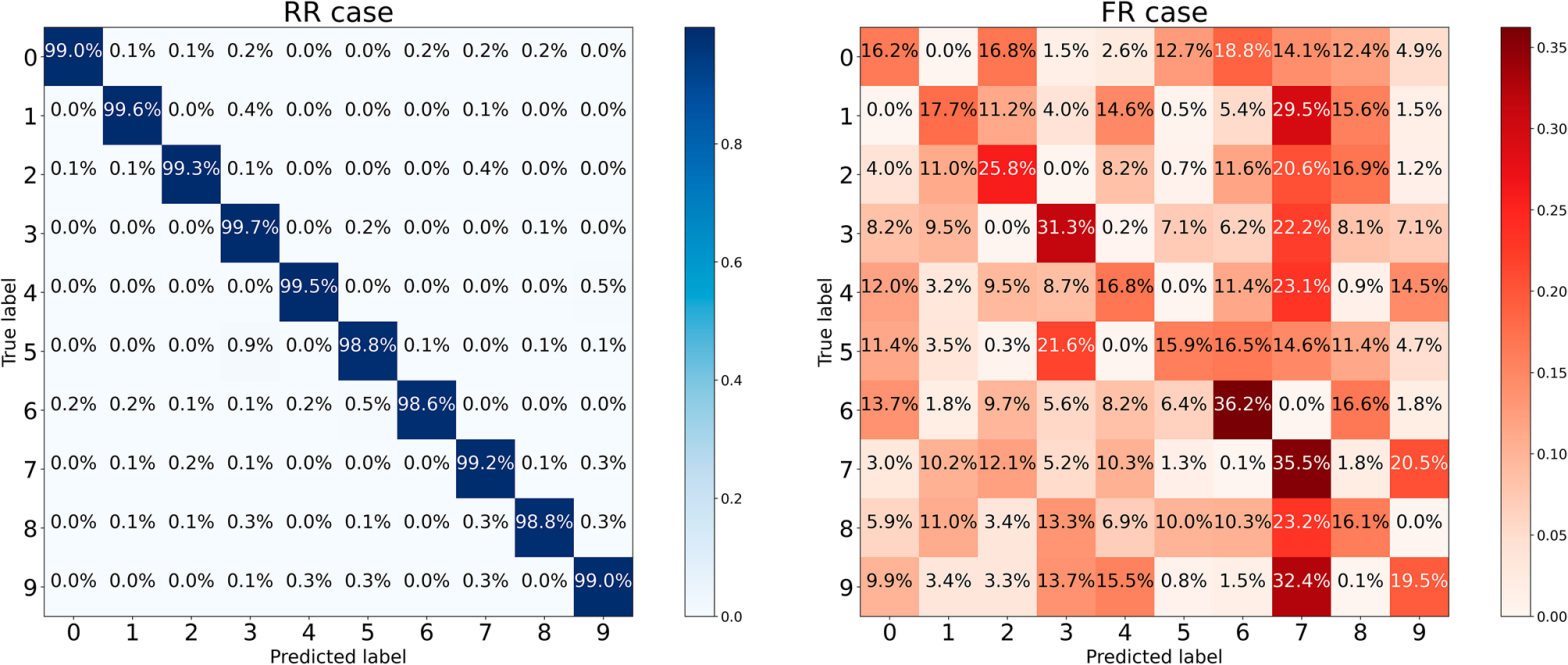
Normalized confusion matrices reporting performances for the task of classifying MNIST database digits with CNNs operationalizing the normotypical and pathological cognitive processes. In the former case (RR, left panel) both the training and the test sets are composed of images in which a color is randomly assigned to digits. In the latter case (FR, right panel) in the training set colors are associated to specific digits according to a fixed prescription, while the test set is composed of images in which a color is randomly assigned to digits.

The normalized confusion matrices reporting the CNN classification performance in RR and FR cases respectively are shown in Fig. 3. The gap between the two cases is visually striking and confirmed by the difference between the values of a global performance quantifier such as accuracy, namely 0.9899 for RR, and 0.2273 for FR. Notice that, in the latter case, the performance is still better than the random baseline (0.1), since the algorithm is still able to perform a good discrimination between digits drawn in the same color in the FC training set.

### Principal component analysis: pixel level

**Figure 4:**
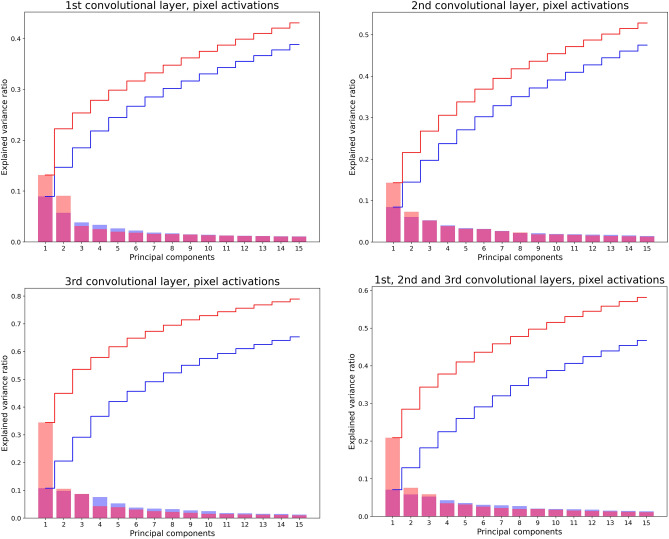
PCA output*—*pixel level. Distribution of the explained variance over the 15 first factorial dimensions for both RR (blue plot) and FR (red plot). Top panels and bottom left panel refer to first, second and third layers respectively; bottom right panel refers to all layers. Histograms correspond to the explained variance of each component; lines represent cumulative distributions. Notice that the bars appear purple in the overlap between the two histograms.

For visualization purposes, Fig. 4 reports the results on the relative importance of the main components of the aforementioned matrices, quantified in terms of the ratio between the related explained variance and the total variance of the PCA output. In each plot, both the individual and the cumulative explained variances are reported. It is evident that, in the FR case, the variance is much more concentrated on the first factorial dimensions. As we can also see from Table 1, the weight of primary dimensions (WPD) is systematically higher for FR, compared with RR. This difference is particularly striking in the third layer, in which WPD for FR corresponds to 44.92% of the explained variance, against the 20.52% of RR. Notably, the situation is reversed for the weight of secondary dimension (WSD; cf. Table 1), which is systematically higher for RR than for FR. Also in this case, this is particularly true in the third layer (50.76% of explained variance in RR *vs.* 36.96% in FR). Table 1 also reports the D90 -i.e. the number of relevant components required to explain 90% of the total variance. PCA findings suggested that the increased importance of the primary dimensions consistently leads to a smaller number of components explaining 90% variance in FR for all layers, but especially for layer 3, where only 44 components were needed versus 95 in RR .

**Table 1:** Dimensionality of the factorial space: pixel level.

	Indexes of dimensionality					
	Weight of primary dimensions (WPD) (% Variance Explained, PC 1 + 2)		Weight of secondary dimensions (WSD) (% Variance Explained, PC after the 2nd with eigenvalue > 1)		Minimal number of components explaining at least 90% variance (D90)	
	RR (%)	FR (%)	RR (%)	FR (%)	RR	FR
Convolutional layer 1	14.67	22.23	35.07	21.82	416	389
Convolutional layer 2	14.46	21.56	47.17	45.51	229	177
Convolutional layer 3	20.52	44.92	50.76	36.98	95	44
All convolutional layers	12.91	28.48	56.09	48.29	348	210

### Principal component analysis: filter level

**Figure 5:**
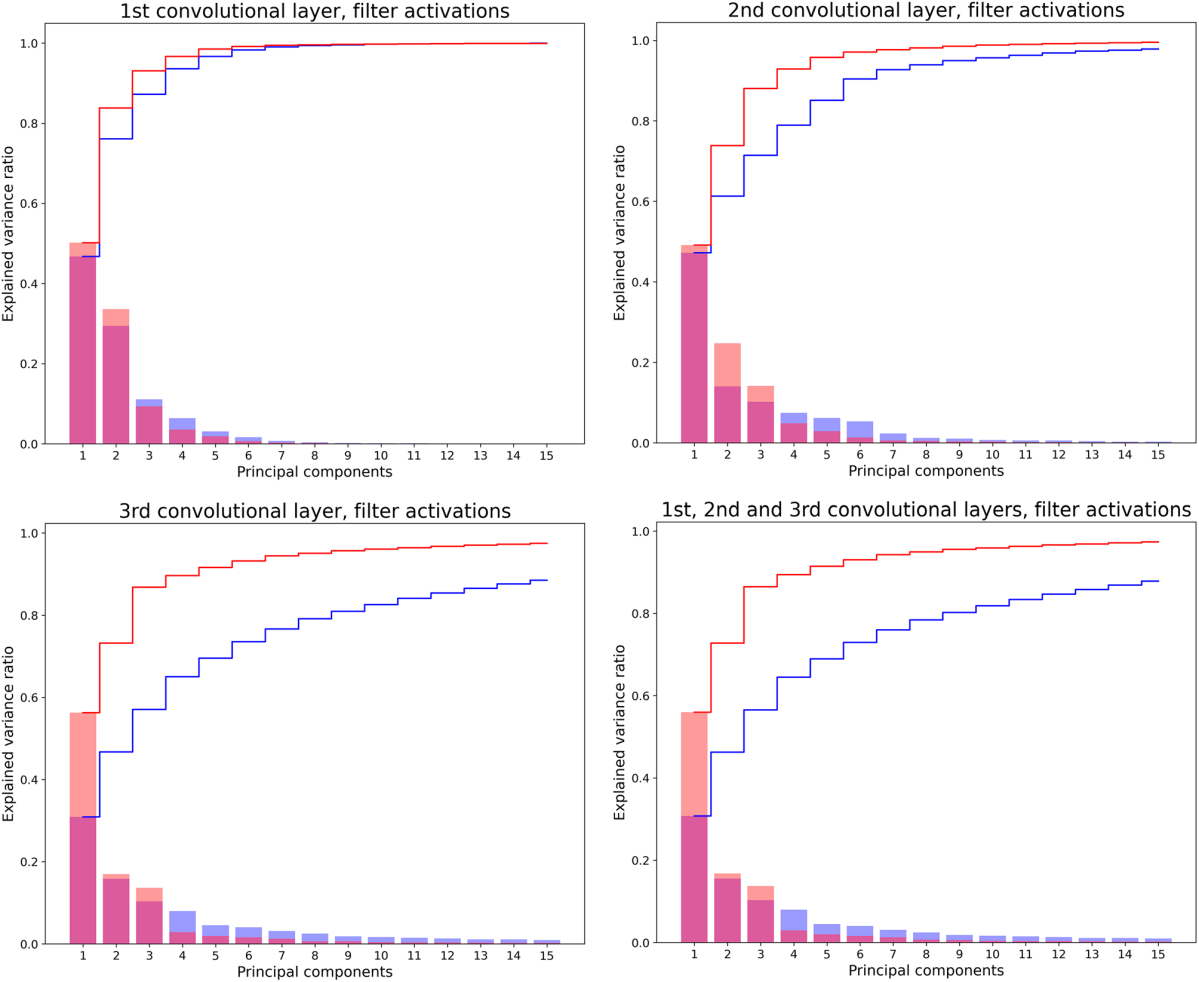
PCA output*—*filter level. Distribution of the explained variance over the 15 first factorial dimensions for both RR (blue plot) and FR (red plot) cases. Top panels and bottom left panel refer to first, second and third layers respectively; bottom right panel refers to all layers. Histograms correspond to the explained variance of each component; lines represent cumulative distributions. Notice that the bars appear purple in the overlap between the two histograms.

The outcomes of the PCA analysis at the filter level, summarized in Fig. 5 for visualization purposes, confirmed the results obtained at the pixel level. Actually, the pivotal role of the third layer in marking a difference between RR and FR cases, already noticed in the previous analyses, emerges in a clearer way by focusing on filters as a whole. As we can see from the investigation of WPD and D90, while in the first and second convolutional layers all outcomes for the RR and FR configurations are comparable, differences become much more relevant when the third layer is considered (Table 2). These differences are then reflected in the overall three-layer PCA: (i) the WPD is quite a bit higher in the FR than in the RR CNN (layer 3: 73.18% in FR vs. 46.72% in RR; overall layer distribution: 72.76% for FR vs. 46.23% for RR); (ii) D90 is quite a bit higher in RR than in FR (layer 3: 5 components in FR vs. 17 in RR; overall layer distribution: 5 components for FR vs. 18 for RR). All findings are reported in Table 2.

**Table 2:** Dimensionality of the factorial space: filter level.

	Index of dimensionality			
	Weight of primary dimensions (WPD) (% Variance Explained, PC 1 + 2)		Minimal number of components explaining at least 90% variance (D90)	
	RR (%)	FR (%)	RR	FR
Convolutional layer 1	76.12	83.78	4	3
Convolutional layer 2	61.26	73.88	6	4
Convolutional layer 3	46.72	73.18	17	5
All convolutional layers	46.23	72.76	18	5

### Two-sample t-tests on the mean activation value distributions

**Figure 6:**
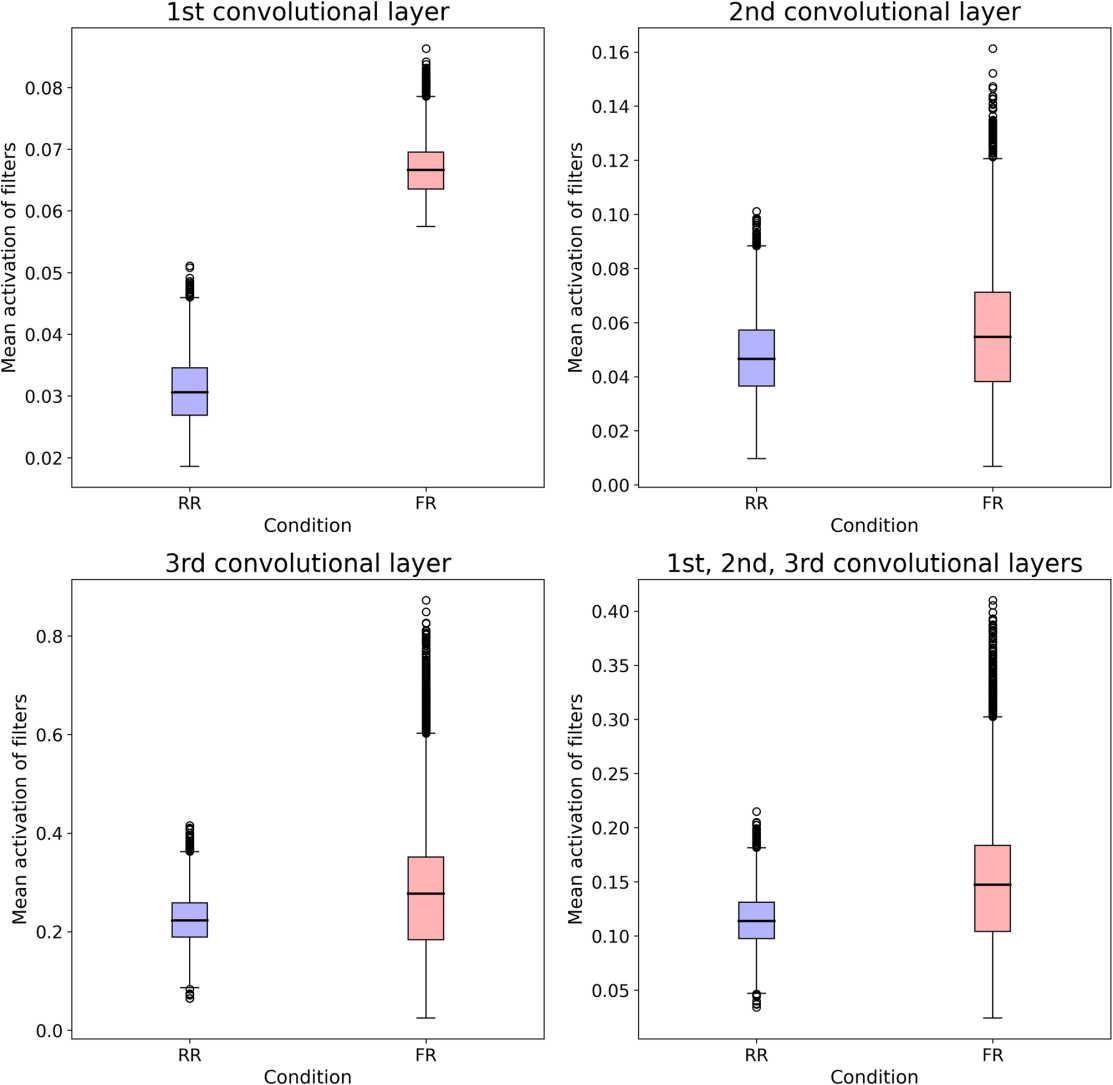
Distributions of mean pixel activation values of filters in each of the three convolutional layers and together. Comparison between RR (blue plot) and FR (red plot).

In all analyses, the FR distribution showed a larger average of the mean pixel activation distribution than its RR counterpart (all p < 0.05, FDR corrected, Table 3), indicating a heavier resource consumption in the former CNN. For the first layer, the two distributions do not overlap; for the second and third layer, as well as when considering all layers together, the overlap is relevant, but the FR distribution is characterized by more pronounced tails, indicating wider fluctuations in the mean activation, with varying test samples, as shown by Fig. 6.

**Table 3:** Main results of the two-sample *t*-test analyses comparing FR vs. RR network’s pixel mean response values across all network filters and their aggregation.

Dependent variable, two sample t-test (FR vs. RR)	*t* value	*p* uncorrected	*p* FDR corrected
Mean response values Layer 1	527.415	< 0.0001	< 0.0001
Mean response values Layer 2	32.528	< 0.0001	< 0.0001
Mean response values Layer 3	40.489	< 0.0001	< 0.0001
Mean response values Layer 1,2,3	51.394	< 0.0001	< 0.0001

## Discussion

Recent literature on the p factor construct has outlined the possibility of a unifying conception of psychopathology. This has led researchers to attempt to identify a potential plausible core mechanism underlying psychopathology. The harmonium model (HM) represents a contribution in this direction, consisting of a computational model of the mechanism underlying psychopathology^41^. According to the HM, psychopathology is a matter of a low-dimensional Phase Space of Meaning, namely a mode of processing the environment focusing on very few of its properties, therefore blind to the nuances and marginal information that enable the individual to build appropriate interpretation of the situation to address.

This study was aimed at providing further, straightforward validation of the HM. To this end, we developed two CNN configurations, simulating normotypical (RR) and psychopathological (FR) cognitive processing, respectively; then we compared the dimensionality of the dynamics of functioning of the two CNN configurations. The two CNN configurations were obtained by varying the complexity of the classification task implemented as training stage—this was done on the grounds of the assumption that the capacity of the CNN to accomplish the classification task in the test stage depends on the complexity of the training state^10^.The preliminary comparison of the accuracies of the FR and RR networks highlighted a striking difference between them. Indeed, without any variation in the CNN architecture implementation, except for the learning rule of association between color and digit adopted in the training stage (fixed for the FR condition, and random for the RR condition), the CNN performed with only 0.2273 accuracy in the FR case, compared with the 0.9899 accuracy achieved in the RR case. This finding supported our assumption that the two CNN configurations were able to simulate a normotypical and a pathological cognitive processing, respectively—operationally measured in terms of performance level (i.e., low vs. high accuracy).

The main finding from this study, which aligns with our hypothesis, is that the accuracy differences between CNNs in FR and RR is mirrored by striking differences in the dimensionality of the dynamics of functioning of the two configurations—and this is so at the level of both single pixels and entire filters. At both levels of analysis investigated—i.e. pixel (Table 1) and filter activations (Table 2), PCA revealed that the RR configuration’s processing needs a higher number of dimensions to be described than that required by the description of the FR configuration. Moreover, in the case of the RR configuration the explained variance is evenly distributed across components, compared with what happens in the FR configuration. Indeed, as shown by Figs. 4 and 5 and Tables 1 and 2, in the FR configuration we found that the amount of explained variance due to the primary dimensions is quite a bit higher than in RR. Also in this case this pattern emerges both at pixel and filter levels. Conversely, the weight of secondary dimensions is quite a bit lower in the FR case than in the RR case (this finding concerns only the pixel level, given that it was not possible to apply the index at the filter-level).

Insofar as one assumes that the two FR and RR configurations are a proxy of psychopathological/normotypical conditions, the difference found in the dimensionality of the two CNN configurations represents a computational validation of the core tenet of the HM—namely of the hypothesis that the cognitive process underlying the psychopathological condition is characterized by a low-dimensional PSM, when compared to a normotypical cognitive process. Thus, the findings of the current simulation study are consistent with the view of psychopathology as due to poorly modulable PSM^10^ which forces the individual to interpret the environment rigidly, in terms of basic components of meaning (i.e. the primary dimensions, according to the HM terminology), and to background more fine-grained components (i.e. the secondary dimensions), those required to detect nuanced, efficacious maps of the circumstances.

According to the HM, the PSM primary dimensions consist of generalized, affect-laden classes of meanings, which provide information regarding the interpretation of experiences as a whole, irrespective of the details of the environmental scenario^55^. These primary dimensions show a very low degree of variability across individuals, thus being mainly invariant^56^. Accordingly, the higher their momentum in the PSM, the more rigid the cognitive process is, operating by means of invariant classes of meaning. On the other hand, in normotypical conditions (represented by the RR CNN configuration), a highly modulable PSM corresponds to a low weight of the primary dimensions, which leaves room for secondary dimensions. The secondary dimensions are information-based and therefore up-to-date with the characteristics of current environmental stimuli^40,41^; they are the components of meaning supporting an effective fit between individual and environment (whose computational proxy is represented by the high RR CNN accuracy).

Interestingly, the differences discussed are mainly visible either when we considered all network layers together, or when we considered the third convolutional layer alone. As outlined in our Materials and Methods section, we have three different convolutional layers within both FR and RR networks, interleaved by two max pooling layers. The layer in which we observed the greatest difference between FR and RR networks is the one immediately preceding the dense output layers, that is, the one farthest away from the test input data. As we have employed a CNN structure with multiple convolutional layers, this structure can be considered somehow hierarchical (that is, later layers operate on information that has been previously processed by prior ones). In other words, each prior layer provides a “lower level” pattern which serves to inform the subsequent level. The differences between CNNs in the RR and FR configurations are maximized at the highest level of these hierarchical patterns^54^. We may thus speculate that the more the network generates rules of learning, layer by layer, the more the cognitive process (pathological vs. normotypical) leads the CNN to rely on rigid (FR), rather than flexible (RR) patterns of meaning. Within our interpretation framework, this computational process very closely resembles the use of heuristics in human cognition and social processes. Heuristics have been previously defined as cognitive strategies, of which individuals may show different degrees of awareness, which are employed to guide behavior in the social environment in presence of insufficient time, information or cognitive resources^57^. They are a means to reduce the complexity and uncertainty of human environments and are employed to yield an easier and faster decision-making process, mainly through the strategy of overestimating few sources of information while ignoring many others. In this sense, heuristics have been previously defined as a form of cognitive bias^58^. Therefore, assuming again that our computational design is a way of operationalizing psychopathological/normotypical conditions—and the underlying PSM dimensionality—the differences between the FR and RR CNNs in terms of factorial components grouping their information variance may be a proxy of the use of rigid (being mainly explained by few sources of information) heuristics which guide behavior towards failure (testified by the low FR CNN accuracy in the test phase). Indeed, in a real-world scenario, the rigid use of heuristics has been previously discussed and consistently associated with a failure in effective decision making^59^, and ultimately, increased risk of psychopathology. Consistently, in the psychological field, rigidity has been defined as *“the tendency to develop and perseverate in particular cognitive or behavioral patterns, and such patterns being continuously employed in situations where the pattern is no longer effective”*^49^. As an example, a person diagnosed with paranoid personality disorder may adopt a rigid interpretative schema whose application leads her/him to see other people as threatening, hostile enemies in almost all circumstances, regardless of the actual signals provided by the latter. As a result, the person will make behavioral and interpersonal choices coherent with the perceived threat (e.g., hyper-vigilance, suspicious control of interpersonal proximity, aggressive counterattack). Conversely, flexibility describes individuals that *“despite having formed a particular cognitive/ behavioral pattern of responding to a specific situation, are able to disengage from this initial pattern if the initial pattern of responding is no longer effective for the specific situation”*^49^*.* A recent review has highlighted that, across studies, different measures of rigidity have been associated with forms of cognitive processing maintaining psychopathology, especially for what concerns the inflexible use of rumination and perfectionism^49^.

Notably, the comparison between the level of pixel activation characterizing the two CNN configurations highlighted a functional characteristic associated with the low dimensionality of the FR network. Indeed, the t-test findings show that the mean pixel activation in all filters, within and across the three convolutional layers, was significantly higher in the FR network than in the RR network. In other words, the FR network had much more intensely activated pixels, within each layer and across all layers, compared with the RR network. In light of the much lower accuracy of the FR network compared with the RR network in the test phase, this finding indicates a waste of computational resources in the FR network, as this higher intensity of pixel response of the FR network did not mirror either an absolutely good performance, or a performance comparable to the one of the RR network.

Thus, one is led to conclude that the low-dimensionality of the FR CNN corresponds to a mode of functioning of the cognitive system characterized by lack of organization—i.e. capacity to provide an efficacious structure to experience. This lack of organization leads to a waste of computational resources, as if the cognitive system were compelled to increase its activity to surrogate the low level of capacity to detect the relevant information from the environment—in brief, the cognitive system works more because it works badly.

In the final analysis, paralleling our computational framework to a real-world framework, we may potentially speculate that ineffective processing, therefore psychopathology, may rely on an excessive amount of resources rigidly directed towards redundant/irrelevant environmental patterns of information, and thus wasted. This waste of resources could potentially crystalize in a rigid heuristics, which would become maladaptive because of its non-finalized, invariant use.

Taken as a whole, the findings of the current simulation study have intriguing implications. The neural network architecture adopted enabled to open the black box of the information processing substantiating how psychopathology works. This supports the HM explanation of the p factor theory of psychopathology. In so doing, this study contributes to take the unifying view of psychopathology a step ahead, moving it from the merely descriptive recognition of the overlaps among psychopathological categories, to the modelling of the computational mechanism underpinning such overlaps. The theoretical and practical relevance of this advance does not need to be underlined. From a theoretical standpoint, psychology, like any science, develops thanks to and in the terms of moving from descriptive to explicative theories, namely, theories that go beyond the identification of empirical linkages between antecedents and consequents by modelling the latent mechanisms underlying such linkages^40^. Moreover, the dimensional model of psychopathology frames a computational interpretation of the psychological clinical change. The distinction between I-order and II order change is quite popular in clinical psychology (e.g. Ref.^60^) and more in general in psychology (e.g. Ref.^61^). However, this distinction is generally based on the characteristics of the manifestations of the change—e.g. in the momentum of the modifications involved. The harmonium model complements such a descriptive approach by providing a computational model of the structure of the cognitive processes underpinning the two levels of change—i.e. I-order change as a variation within the given PSM dimensionality; II-order change as a variation of the PSM dimensionality^41^. Finally, the development of computational models of the mechanisms of psychopathology has a high practical value too. Indeed, the more one understands the mechanisms of psychopathology, the more one can develop strategies to counteract it—as well as methods and settings to train professionals.

A further implication of the study is worth noting. As observed above (Aims section), the present study establishes the psychopathological network by manipulating the dimensionality of the training task, consistently with the HM framework. This methodological choice paves the way to a further development of the model, in the direction of a dimensional theory of the genesis of psychopathology. We see the chance to design future studies based on a reverse engineering approach—namely, to assume that the psychopathological cognitive processing is characterized by the network’s low-dimensional behavior and, on these grounds, to focus on the characteristics of the training stage producing such a low-dimensional behavior.

Before concluding, it is worth mentioning the limitations of the study. Although our findings extend those previously published by our group^10^, the current investigation is based on only one task (namely, digit recognition) and two training conditions (i.e., FR and RR). To understand the validity of our findings more in depth, replication in other tasks, and with other sources of information (e.g., more complex pictures, rather than numbers, or even images depicting real-world stimuli) under more than two training conditions, is certainly needed. Furthermore, it is important to point out that the discussion of our findings relies on the assumption that the joint investigation of CNN accuracies and their internal computational dynamics is a proxy of psychopathological/normotypical conditions and PSM dimensionality, respectively. Therefore, though we were able to generate a computational framework for psychopathology, this does not entitle us to conclude that this is the only possible mechanism of psychopathology. Conversely, our findings should be taken as a further validation of the HM and of the PSM as a reliable framework to explain one potential mechanism underlying psychopathology and a call for future studies aimed at systematically validating our findings under real-world conditions to fully understand the mechanisms and modulators of the HM.

## Data Availability

The datasets generated and/or analysed during the current study are either publicly available on databases cited in the bibliography, or available from the corresponding author on reasonable request.
